# Role of Nutrient-Sensing Signals in the Pathogenesis of Diabetic Nephropathy

**DOI:** 10.1155/2014/315494

**Published:** 2014-07-14

**Authors:** Shinji Kume, Daisuke Koya, Takashi Uzu, Hiroshi Maegawa

**Affiliations:** ^1^Department of Medicine, Shiga University of Medical Science, Tsukinowa-Cho, Seta, Otsu, Shiga 520-2192, Japan; ^2^Division of Diabetology & Endocrinology, Kanazawa Medical University, Kahoku-Gun, Ishikawa 920-0265, Japan

## Abstract

Diabetic nephropathy is the leading cause of end-stage renal disease worldwide. The multipronged drug approach still fails to fully prevent the onset and progression of diabetic nephropathy. Therefore, a new therapeutic target to improve the prognosis of diabetic nephropathy is urgently required. Nutrient-sensing signals and their related intracellular machinery have evolved to combat prolonged periods of starvation in mammals; and these systems are conserved in the kidney. Recent studies have suggested that the activity of three nutrient-sensing signals, mTORC1, AMPK, and Sirt1, is altered in the diabetic kidney. Furthermore, autophagy activity, which is regulated by the above-mentioned nutrient-sensing signals, is also altered in both podocytes and proximal tubular cells under diabetic conditions. Under diabetic conditions, an altered nutritional state owing to nutrient excess may disturb cellular homeostasis regulated by nutrient-responsible systems, leading to exacerbation of organelle dysfunction and diabetic nephropathy. In this review, we discuss new findings showing relationships between nutrient-sensing signals, autophagy, and diabetic nephropathy and suggest the therapeutic potential of nutrient-sensing signals in diabetic nephropathy.

## 1. Introduction

**Figure 1:**
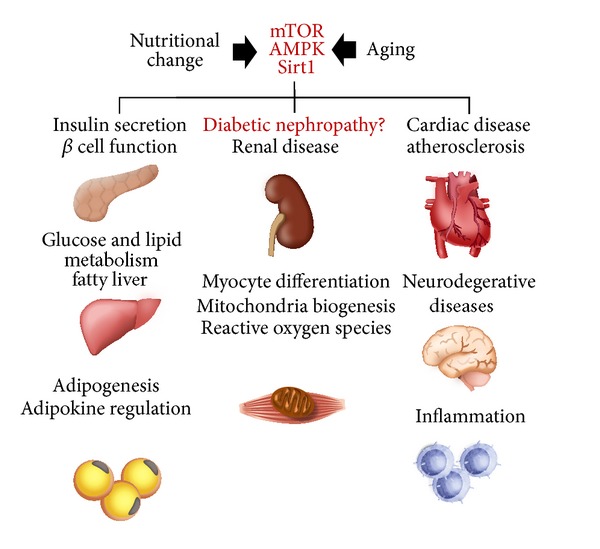
Nutrient-sensing signal and diseases. The three nutrients-sensing pathways, mTOR, AMPK, and Sirt1, independently and coordinately regulate organ metabolism in multiple organs. Their alterations are involved in the pathogenesis of obesity-related and age-related diseases.

Nutrient-sensing pathways dependent on extracellular nutrient conditions are well conserved among eukaryotes from yeasts to mammals. Recently, the importance of three nutrient-sensing pathways in metabolism development has become clear as the study of diabetes and obesity advances (Figure 1). Generally, in excessive nutrient conditions, the mammalian target of rapamycin (mTOR) is activated by increases in glucose, amino acid, and insulin levels [1, 2]. However, in nutrient-depleted conditions, AMP-activated protein kinase (AMPK) and oxidized NAD- (NAD^+^-) dependent histone deacetylase (Sirt1) are activated by increases in intracellular AMP and NAD^+^ levels, respectively [3–5]. These nutrient-sensing pathways use posttranslational phosphorylation and acetylation modification of proteins to regulate energy homeostasis in metabolic organs under both excess and restricted nutrient conditions [1–5]. Dysregulation of these pathways is associated with the development of diabetes [1–5].

Interestingly, these pathways also exist in the kidney [6–9], and tissue concentrations of glucose, amino acid, AMP, and NAD^+^ are modified by particular nutrient states, such as diabetes, and diet therapies, such as protein or dietary restriction. These findings led us to consider whether these signaling pathways are involved in the pathogenesis of renal diseases and whether they may be potential therapeutic targets for treating renal diseases associated with diabetes and obesity [9].

In the multiple organs involved in diabetes and obesity, cells experience stress with nutrient excesses, leading to organelle dysfunction, including mitochondrial stress [10], peroxisomal oxidative stress [11, 12], and endoplasmic reticulum (ER) stress [2], which are thought to be pathological factors in diabetic nephropathy [11–14]. We considered how it might be possible to protect organelles against stresses mediated by excessive nutrient conditions.

The study of “autophagy” in mammalian systems is advancing rapidly, and many researchers are entering this new and exciting field. Autophagy plays a critical role in removing damaged organelles for the maintenance of intracellular homeostasis [15]. Interestingly, this process is also regulated by the above-mentioned nutrient-sensing pathways [16, 17].

In this paper, we review the roles of nutrient-sensing pathways in renal diseases associated with diabetes and obesity and provide a perspective that may assist future research in this field.

## 2. mTOR in Diabetic Nephropathy

Of the three nutrient-sensing pathways, the pathological roles of mTOR and the therapeutic potential of rapamycin, an inhibitor of mTOR, in diabetic nephropathy are increasingly being examined in experimental animal models (Table 1).

mTOR is a nutrient-sensing signal which was first identified in yeast mutants resistant to the effects of rapamycin, an immunosuppressive agent. mTOR associates with the nonenzymatic regulatory-associated scaffold proteins of TOR in mTOR complex 1 (mTORC1) and the rapamycin-insensitive companion of mTOR in mTOR complex 2 (mTORC2) [1]. Rapamycin can inhibit the function of predominantly mTORC1 but not mTORC2.

Activation of mTORC1 most prominently results in phosphorylation of two downstream targets, ribosomal S6 kinase and eukaryotic translation-initiation factor 4E-binding protein [1]. These proteins stimulate ribosome biogenesis and protein translation, leading to increases in cell mass [1]. However, current information on the exact role and regulatory mechanisms of mTORC2 is still limited. Thus, knowledge of the role of mTOR in diabetic nephropathy is largely limited to the role of mTORC1.

Over the past few years, the role of mTOR in the pathogenesis of diabetic nephropathy has received increased attention. Studies using rapamycin were important in defining the role of the mTORC1 pathway. After being investigated primarily for its antiproliferative effects, it was increasingly clear that the mTOR pathway has broad implications in both normal and diseased physiologies, including regulating cell and organ size [1]. Additionally, the role of rapamycin has been studied in a wide spectrum of kidney diseases, including diabetic nephropathy [6, 27]. It has long been known that typical features of diabetic nephropathy include renal and glomerular hypertrophy, proteinuria, and advanced fibrosis and inflammation. In addition to inhibiting renal and glomerular hypertrophy present in the early stages of diabetic nephropathy [20], rapamycin seems to ameliorate mesangial expansion, glomerular basement membrane thickening, renal fibrosis and macrophage recruitment, and the development of proteinuria in diabetic rodent models [18, 19, 21–25] (Table 1). These findings suggest that mTOR activation may contribute to the pathogenesis of typical lesions, such as glomerular sclerosis and renal hypertrophy, in diabetic nephropathy.

Two recent studies using heterozygous-raptor knockout mice revealed that hyperactivation of the mTORC1 signal is strongly associated with the progression of podocyte injury and proteinuria in diabetic animal models [27, 28]. In the podocyte of heterozygous-raptor knockout mice showed a resistance to diabetes-induced dysregulation of foot process formation and apoptosis leading to podocyte loss. Furthermore, we recently reported that obesity-mediated hyperactivation of the mTORC1 signal led to the suppression of autophagy activity in proximal tubules resulting in the development of cell vulnerability, which were ameliorated by calorie restriction and rapamycin [26]. These findings suggest that mTORC1 activation in all kinds of renal component cells may be involved in the pathogenesis of diabetic nephropathy.

mTOR is activated by both insulin and nutrients such as glucose and amino acids in renal cells in diabetic and obese states. Because hyperglycemia occurs along with hyperinsulinemia in obese type 2 diabetic patients, combined hyperinsulinemic and hyperglycemia would be expected to activate the mTOR pathway in obese and type 2 diabetic patients. However, hyperglycemia, rather than growth factors such as insulin, may contribute more to mTOR activation in diabetes because mTOR is activated even in the kidneys of insulin-independent type 1 diabetic models [6].

In summary, mTOR induces the synthesis of matrix proteins associated with basement membrane thickening and mesangial matrix accumulation. Additionally, mTOR enhances fibrosis through fibroblast proliferation, epithelial-to-mesenchymal transition, and the expression of profibrotic cytokines such as transforming growth factor-*β*1 and connective tissue growth factor. mTOR-dependent infiltration of macrophages and production of proinflammatory cytokines, such as MCP-1, might support inflammation during diabetic nephropathy [18, 19, 21–25]. Additionally, hyperactivity of mTORC1 signal is strongly related to podocyte dysfunction characterized by dysregulation of nephrin protein and podocyte loss [27, 28]. Finally, more recent reports demonstrated that mTOR contributes to proximal tubular cell apoptosis and damage in diabetes [26, 29]. Thus, it is evident that mTOR plays a central role in the development of the major features of diabetic nephropathy, although additional mechanisms are likely to be involved.

Caution is necessary when interpreting the role of mTOR in diabetic nephropathy because many of the current insights regarding mTOR are derived from pharmacological inhibition of mTOR by rapamycin [30]. Additionally, as mentioned above, our understanding of the regulatory mechanisms and substrates of mTORC2 is very limited. Thus, kidney tissue-specific analysis of mTORC1- and mTORC2-dependent signaling in mice is required to further understand the functional role of mTOR in diabetic nephropathy.

## 3. AMPK in Kidney Diseases

AMPK is a ubiquitously expressed kinase that also acts as an energy sensor [3]. Kinase activity is strictly regulated by extracellular nutrient conditions, which are detected as rising concentrations of AMP, and increases in AMP/ATP ratios [3]. Additionally, AMPK activity is positively regulated by an adipokine, adiponectin [31]. During energy-depleted conditions, there is a marked increase in both intracellular concentrations of AMP and plasma adiponectin levels, leading to activation of AMPK. Activated AMPK acts to restore energy homeostasis by phosphorylating multiple substrates to enhance energy production and suppress energy consumption [3]. In metabolic organs such as skeletal muscle, brown adipose tissue, and the liver, AMPK enhances lipolysis through phosphorylation of acetyl-CoA carboxylase (ACC) and mitochondrial biogenesis and suppresses hepatic gluconeogenesis [3]. Thus, AMPK acts as a nutrient sensor to maintain cell and tissue homeostasis under energy-depleted conditions.

Several studies on whole renal cell lysates have demonstrated that AMPK expression and activity in the kidney are determined by phosphorylation at Thr^172^ [32–36]. Immunoelectron microscopy analysis of its exact localization in glomeruli revealed that AMPK is expressed in podocytes, mesangial cells, and glomerular endothelial cells, although its expression pattern in different renal tubular epithelial cells remains uncertain.

Recently, our understanding of the pathophysiological role of AMPK in the kidney has increased [8]. The expression and activity of AMPK have been examined in several types of diabetic rodent models (Table 2). In streptozotocin- (STZ-) induced type 1 diabetic models, AMPK expression and activity decreased [32–34]. The exact mechanism underlying the decrease in renal AMPK activity in this model is unknown; however, it is possible that decreases in plasma adiponectin, in addition to hyperglycemia, could contribute to this decrease [33]. Pharmacological activation of AMPK by 5-aminoimidazole-4-carboxamide-1-*β*-d-ribofuranoside (AICAR) and metformin ameliorated renal hypertrophy in STZ-induced diabetic rats [34]. In diet-induced obese mouse models and type 2 diabetic db/db mice, renal AMPK activity was suppressed and plasma adiponectin levels were reduced [35, 36]. Interestingly, adiponectin was able to stimulate AMPK activity in podocytes, mesangial cells, and glomerular endothelial cells [39], and adiponectin-deficient mice showed development of albuminuria together with decreased AMPK activity in podocytes [38]. These findings suggest that activation of the adiponectin-AMPK pathway may be a therapeutic target for diabetes- and obesity-related kidney diseases.

It is likely that AMPK activity decreases in the kidneys of diabetic and obese animals, which raises the question of how decreases in AMPK activity are involved in the pathogenesis of renal diseases. A study has reported that, in the kidney of obese mice, intrarenal lipid metabolism was altered and was characterized by enhanced renal lipogenesis and suppressed lipolysis [36]. AMPK-mediated phosphorylation inactivates a lipogenic enzyme, ACC, which results in decreased lipogenesis and enhanced lipolysis [3]. Decreases in kidney AMPK activity in these mouse models may be involved in altered renal lipid metabolism and subsequent lipotoxicity-associated renal damage. Recent reports suggest that altered mitochondrial biogenesis and subsequent ROS production under excessive nutrient conditions contribute to the pathogenesis of metabolic diseases such as diabetes and obesity. Proximal tubular cells contain a large number of mitochondria because the energy demand in these cells is relatively higher than those of other cells. Thus, the altered mitochondrial biogenesis observed under diabetic conditions might be associated with ROS production and subsequent tubular damage [35]. Collectively, decreases in renal AMPK activity may cause the typical features of renal disease in obese and diabetic patients. Actually, pharmacological activation of AMPK by AICAR significantly improved renal injury including albuminuria in diabetic Akita mice [37].

## 4. Sirt1 in Kidney Disease

Calorie restriction slows aging and increases life span in different species. Even if calorie restriction is shown to increase life expectancy in humans, it is unlikely that such restrictions will be widely adopted because of the difficulty in maintaining long-term calorie restriction in modern society. Therefore, there has been increased interest in identifying molecules that act as “calorie restriction mimetics.” Interestingly, several recent studies have revealed that Sirt1, one of seven mammalian sirtuin/Sir2 genes in the NAD^+^-dependent deacetylase family, could be a common mediator that explains the health benefits under calorie restriction [4, 5]. Furthermore, several recent reports have shown that Sirt1 regulates several biological functions, including cell survival, mitochondrial biogenesis, insulin secretion, and glucose and lipid metabolism in various tissues [5].

Reduced forms of nicotinamide adenine dinucleotide (NADH) are metabolites of glucose and fatty acids. Thus, NAD^+^/NADH ratios decrease in cells under nutrient excess. Sirt1 senses intracellular NAD^+^ levels and acts as a deacetylase in a NAD^+^-dependent manner. Protein acetylation can be regulated in a metabolically responsive manner, and this process may also contribute to adaptation to metabolic stress. Collectively, these findings suggest that Sirt1's deacetylase activity decreases in nutrient excess conditions, and its activation, which acts as a calorie restriction mimetic, may be a new therapy for renal disease in obese and diabetic patients. However, the relationship between Sirt1 and diabetic complications is not well known. We discuss here the role of Sirt1, and speculate on the possible involvement of Sirt1, in renal diseases in patients with diabetes and obesity.

The effects of nutrient conditions on Sirt1 expression levels in the kidney have been determined (Table 3). Dietary restriction in rats increases Sirt1 expression, which is associated with decreases in plasma growth factors such as insulin/IGF-1 [42]. In STZ-induced type 1 diabetic kidneys, Sirt1 expression decreases [40, 41], but Sirt1 expression does not change in kidneys of type 2 diabetic db/db mice [35]. Sirt1 activity is strictly regulated by intracellular NAD^+^ concentrations [50], which are likely to decrease in diabetic organs. Thus, to determine whether Sirt1 activity is altered in diabetic kidneys, it is necessary to assess the deacetylation of Sirt1 substrates such as NF*κ*B (p65), p53, and forkhead proteins [4].

Many researchers are entering this exciting field, and interesting findings showing the renoprotective effects of Sirt1 have been reported (Table 3). Sirt1 expression and activity determined by deacetylase activity on the Foxo3a substrate were significantly altered in aging kidneys [16]. Furthermore, heterozygous Sirt1-deficient mice showed dietary restriction-resistant premature renal aging [16]. This evidence suggests that Sirt1 may be involved in renal aging and dietary restriction-mediated renoprotection.

Tubulointerstitial fibrosis is a common final pathway for end-stage renal diseases, including diabetic nephropathy. Several recent reports have shown the antifibrotic effects of Sirt1 in mouse experimental models [44, 46]. In those reports, mechanisms of Sirt1's antifibrotic effects were explained by suppression of both cyclooxygenase 2 expression [44] and activation of TGF*β*-induced Smad3 [46], both of which are recognized as classical pathogenic factors in diabetic nephropathy. Additionally, in vivo studies by Hasegawa et al. showed direct renoprotective effects of Sirt1 overexpression in proximal tubular epithelial cells (PTEC) [45]. Proximal tubular cells with specific Sirt1 overexpression were resistant to cisplatin-mediated PTEC damage because of overexpression of catalase and subsequent peroxisome protection [45]. Funk et al. showed that the specific Sirt1 activator, SRT1720, enhanced mitochondrial biogenesis through deacetylation of PGC1 and subsequently reduced ROS in cultured proximal tubular cells [49]. The role of Sirt1 in glomerular cells is still small. In cultured mesangial cells, Sirt1 showed antiapoptotic effects against ROS and TGF*β* [47, 48].

Based on more recent reports, Sirt1 activity is likely to be suppressed in the kidney of diabetic animal models [43, 51]. Interestingly, Hasegawa et al. reported that Sirt1 activity in both proximal tubular cells and podocytes was associated with diabetic nephropathy [43]. Interestingly, proximal tubular cell-specific Sirt1 transgenic mice showed a resistance to diabetes-related progression of podocyte damage and subsequent proteinuria [43]. Collectively, these results strongly support Sirt1 activation being considered as a new therapy to improve the prognosis of diabetic nephropathy.

## 5. Organelle Dysfunction and Renal Disease in Diabetes and Obesity

**Figure 2:**
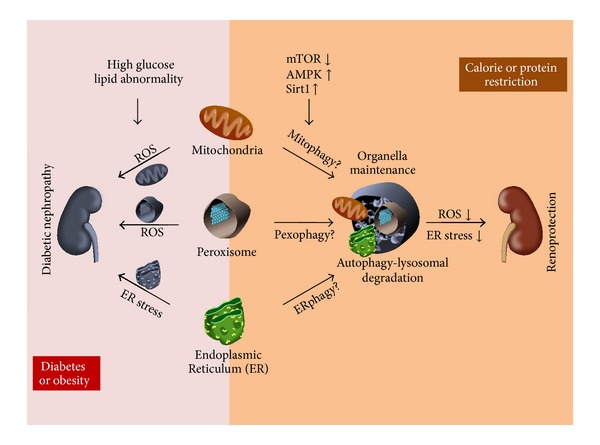
Organelles, such as mitochondria, peroxisome, and ER, dysfunction causes accumulation of reactive oxygen species (ROS) and ER stress in diabetic kidney. Dietary restriction enhances autophagy-lysosomal degradation system, leading to cell or tissue homeostasis. Nutrient-sensing signal and organella maintenance in diabetic nephropathy (DN).

In addition to the classical pathogenesis of diabetic nephropathy, organelle dysfunction, such as in mitochondria [35], peroxisomal oxidative stress [11], and ER stress [13], has been proposed as a theory to explain the emergence of pathological features in diabetic nephropathy (Figure 2). ROS are an inevitable by-product of mitochondrial and peroxisomal metabolism. And excess ROS is implicated with classical pathogenesis by initiating events in diabetic nephropathy [11–14, 35]. In nutrient excess conditions, such as diabetes and obesity, high glucose levels enhance the generation of mitochondrial ROS, and high plasma levels of free fatty acids can also cause ROS overproduction from mitochondria and peroxisomes. Therefore, maintaining healthy populations of functional mitochondria and peroxisomes in different renal cells under nutrient excess conditions is essential for the well-being of cells. This maintenance may prevent the development of renal disease in cases of diabetes and obesity.

ER contains quality-control mechanisms to handle misfolded proteins, and thus ER stress refers to physiological or pathological states that result in the accumulation of misfolded proteins. ER stress in renal pathophysiology is a relatively new area of research in proteinuric kidney diseases, including diabetic nephropathy. Hyperglycemia and high levels of free fatty acids cause ER stress in podocytes [52], which undergo apoptosis and the subsequent initiation of proteinuria in diabetic nephropathy. Additionally, in proteinuric kidney diseases, including overt stages of diabetic nephropathy, proteinuria filtered from glomeruli enhances ER stress responses in proximal tubules, leading to progression of tubulointerstitial lesions [53]. Thus, maintaining the ER's capacity for handling misfolded proteins in podocytes and tubular cells could be considered a new therapeutic target for protecting kidneys from proteinuria-related pathologies.

If this is the case, it remains to be determined how to protect organelles from stresses derived from nutrient excesses. Autophagy is an intracellular process for the degradation of proteins and organelles via lysosomes for the control of cell homeostasis [15]. This degradation system maintains cell homeostasis under nutrient-depleted conditions and has been generally well conserved among all types of eukaryotes. Nutrient excesses inactivate autophagy, but once nutrients are depleted, autophagy is activated to provide energy resources for cells. Interestingly, autophagy is closely regulated by nutrient-sensing pathways and by cellular stresses [16, 17, 54, 55]. Inhibition of mTOR and activation of AMPK and Sirt1 activate nonselective autophagy in response to nutrient depletion [16, 17]. Furthermore, oxidative stress derived from mitochondria and peroxisomes or ER stress can cause autophagy to degrade the damaged organelle itself (organelle-selective autophagy: mitophagy, pexophagy, and ERphagy) [2, 54, 56].

Recent reports have shown that autophagy deficiencies under nutrient excess conditions are related to the pathogenesis of obesity- or aging-associated diseases [15]. Currently, nephrologists are also entering this field of study. Although direct evidence showing the relationship between autophagy and diabetic nephropathy is unfortunately still weak, several interesting findings regarding autophagy have been recently demonstrated in the field of nephrology. Age-associated declines in autophagy in podocytes and proximal tubular cells are part of the development of key features in renal aging, albuminuria, glomerulosclerosis, and ROS production as a result of the accumulation of damaged mitochondria in proximal tubular cells [16, 57, 58]. Interestingly, Sirt1-mediated activation of autophagy under hypoxic conditions in proximal tubular cells is essential for dietary restriction-mediated antirenal aging [16]. Additionally, autophagy was activated in a renoprotective manner in ischemic-reperfusion and cisplatin-induced kidney injury mouse models [58–61]. Thus, autophagy is required for maintaining homeostasis in podocytes and proximal tubular cells and is activated under stress conditions to protect renal cells.

If autophagy is inactivated by mTOR activation and AMPK and Sirt1 inactivation in obesity and diabetic renal diseases, autophagy deficiency could enhance organelle dysfunction mediated by nutrient excess, hypoxia, and proteinuria. Organelle degradation systems regulated by nutrient conditions may be involved in the pathogenesis of renal diseases associated with obesity and diabetes. We previously found that autophagy activity was suppressed in an mTORC1-dependent manner in proximal tubular cells of obese mice, leading to more severe proteinuria-induced tubular interstitial lesions [26].

Autophagy could be a new therapeutic strategy for the treatment of diabetic nephropathy, although solid evidence of the involvement of autophagy in the pathogenesis of podocyte injury is lacking.

## 6. Diet Therapy in Renal Diseases in Diabetes and Obesity

Diet or calorie restriction is essential for strict glycemic control in diabetic patients without advanced nephropathy. Based on results from a large number of experimental studies on diabetes and obesity, ameliorating the above-mentioned nutrient pathways could be a mechanism underlying dietary restriction-mediated improvement of obesity and diabetes. Additionally, recent human clinical studies have shown that the quality of diet affects the development of insulin resistance and new onset of diabetes. A high fructose intake rather than glucose [62], and ingestion of high concentrations of plasma branched-chain amino acids [63], and saturated fatty acids rather than polyunsaturated fatty acids [64] are independent risk factors for developing insulin resistance and diabetes.

In patients with diabetic nephropathy, diet therapies, such as protein restriction, have been used. However, the efficacy of protein restriction is now controversial. If altered nutrient-sensing pathways, lipotoxicity, and organelle dysfunction in kidneys under excess nutrient stresses are involved in the pathogenesis of renal diseases, a high energy intake along with a low-protein diet may cause an insufficiency of protein restriction in the treatment of diabetic nephropathy. It may, therefore, be time to reconsider whether energy excess along with protein restriction is really beneficial. Also, we need to identify what types of carbohydrates, amino acids, and fatty acids in different diet regimens lead to a better prognosis in renal diseases associated with diabetes and obesity. Amelioration of nutrient-sensing pathways altered in the kidney of diabetic and obese animals may be a possible experimental target to examine the beneficial effects of diet therapy.

## 7. Concluding Comments

Currently, the incidence of obesity and diabetes associated with nutrient excess is increasing worldwide. Accordingly, diabetic nephropathy is the leading cause of end-stage renal disease, despite the application of various intensive therapy programs, such as hypoglycemic and antihypertensive therapy. The identification of additional new therapeutic targets for the prevention of renal diseases associated with diabetes and obesity is urgently needed.

This paper has provided a perspective on whether nutrient-sensing pathways are involved in the pathogenesis of diabetic nephropathy and whether they are acceptable new therapeutic targets. In the next few years, additional studies in conditional knockout mice, transgenic mice, and new disease models will elucidate this possibility. These studies will ultimately provide a clearer perspective on whether nutrient-sensing pathways and organelle maintenance by autophagy should be considered novel therapeutic targets for the treatment of renal diseases in diabetes and obesity.

## Figures and Tables

**Table 1:** The roles of mTOR (mTORC1) in diabetic nephropathy.

Experimental type	Renal outcome/phenotype	Mechanism	Reference
S6 Kinase 1^−/−^ mice	Renal hypertrophy↓	Inhibition of p70S6 kinase	Chen et al. [18]
Rapamycin (db/db mice)	Renal and glomerular hypertrophy↓	eEF2 kinase phosphorylation and laminin *β*1 expression	Sataranatarajan et al. [19]
Rapamycin (STZ-diabetic mice)	Renal hypertrophy↓	Inhibition of p70S6 kinase	Sakaguchi et al. [20]
Sirolimus (STZ-diabetic rats)	Glomerular hypertrophy↓, podocyte loss↓	Decreases of TGF-*β* and VEGF expression	Wittmann et al. [21]
Rapamycin (db/db mice)	Albuminuria↓, glomerular lesion↓	Inhibition of p70S6 kinase	Mori et al. [22]
Rapamycin (STZ-diabetic rats)	Albuminuria↓, glomerular lesion↓, and inflammation↓	Decreases of TGF-*β*, VEGF, and MCP-1 expression	Yang et al. [23]
Rapamycin (STZ-diabetic rats)	Albuminuria↓, glomerular lesion↓	Decreases of TGF-*β*, CTGF, and *α*-SMA expression	Lloberas et al. [24]
Gas6^−/−^ mice	Glomerular hypertrophy↓, mesangial expansion↓	Inhibition of akt and p70S6 kinase	Nagai et al. [25]
Rapamycin (HFD-induced obesity)	Proximal tubular cell damage↓	Increase of autophagy activity	Yamahara et al. [26]
Podocyte-specific raptor-heterozygous mice (db/db mice)	Albuminuria↓, glomerular lesion↓	Inhibition of mislocalization of nephrin	Inoki et al. [27]
Podocyte-specific raptor-heterozygous mice (STZ-diabetic mice)	Albuminuria↓, glomerular lesion↓	Inhibition of podocyte loss	Godel et al. [28]

STZ; streptozotocin, PTECs: proximal tubular epithelial cells, TGF*β*: transforming growth factor *β*, VEGF: vascular endothelial growth factor, MCP-1: monocyte chemoattractant protein-1, CTGF: connective tissue growth factor, *α*-SMA: *α*-smooth muscle actin, and PARP: p(ADP-ribose) polymerases.

**Table 2:** The activity and pathophysiological roles of AMPK in kidney disease.

Experimental type	AMPK*α* activity and Renal outcome	Mechanism	Reference
Diabetic models			
STZ-diabetic rats	AMPK*α* expression↑, AMPK*α* activity↓	Unclear	Cammisotto et al. [32]
STZ-diabetic rats	AMPK*α* activity↓	Plasma adiponectin↓	Guo and Zhao [33]
Treatment of AICAR and metformin (STZ-diabetic rats)	AMPK*α* activity↑, renal hypertrophy↓	Unclear	Lee et al. [34]
Db/db mice	AMPK*α* activity↓	Unclear	Kitada et al. [35]
High-fat diet-induced obese mice	AMPK*α* activity↓, renal lipogenesis↑	Unclear	Kume et al. [36]
Treatment of AICAR	AMPK*α* activity↑, Albuminuria↓, and glomerular lesion↓	Improvement of mitochondria dysfunction	Dugan et al. [37]
Nondiabetic models			
Adiponectin^−/−^ mice	AMPK*α* activity↓ in podocytes	Adiponectin deficiency	Sharma et al. [38]
Treatment of adiponectin and AICAR	AMPK*α* activity↑ (podocytes, mesangial cells, and glomerular endothelial cells)	Adiponectin receptor-dependent	Cammisotto and Bendayan [39]

STZ; streptozotocin, AICAR: 5-aminoimidazole-4-carboxamide-1-*β*-d-ribofuranoside.

**Table 3:** The activity and pathophysiological roles of Sirt1 in kidney disease.

Experimental type	Renal outcome/phenotype	Mechanism	Reference
Activity/expression			
STZ-diabetic rats	Sirt1 expression**↓**	Unclear	Tikoo et al. [40]
STZ-diabetic rats	Sirt1 expression↓	Unclear	Li et al. [41]
Db/db mice	Sirt1 expression→	Unclear	Kitada et al. [35]
Calorie-restricted rats	Sirt1 expression↑	Insulin/IGF-1↓	Cohen et al. [42]
STZ- and db/db mice	Sirt1 expression↓	NMN depletion	Hasegawa et al. [43]
Pathophysiological roles			
Sirt1^+/−^ mice (PTECs)	Renal aging↑	Autophagy deficiency	Kume et al. [16]
Sirt1^+/−^ mice (Medullary cells)	UUO-induced renal fibrosis↑	Decrease of Cox2 expression	He et al. [44]
PTECs-specific Sirt1-TG mice	ROS- and cisplatin-induced PTECs damage↓	Increase of catalase expression	Hasegawa et al. [45]
Treatment with resveratrol	UUO-induced fibrosis↓	Suppression of TGF*β*-Smad3 pathway	Li et al. [46]
Sirt1 overexpression (mesangial cells)	ROS-induced apoptosis↓	Inactivation of p53	Kume et al. [47]
Sirt1 overexpression (mesangial cells)	TGF*β*-induced apoptosis↓	Inactivation of Smad7	Kume et al. [48]
Treatment of SRT1720 (PTECs)	Mitochondrial biogenesis↑, ROS↓	Activation of PGC-1*α*	Funk et al. [49]
PTECs-specific Sirt1-TG mice	Diabetes-induced podocyte injury↓	Epigenetic mechanism	Hasegawa et al. [43]

STZ; streptozotocin, PTECs: proximal tubular epithelial cells, ROS: reactive oxygen species, UUO: unilateral ureteral obstruction, TGF*β*: transforming growth factor *β*, IGF-1: insulin-like growth factor 1, Cox2: cyclooxygenase 2, PGC-1*α*: peroxisome proliferator-activated receptor *γ* coactivator-1*α*, and NMN; nicotinamide mononucleotide.
